# The Mechanism of Regulated Release of Lasso/Teneurin-2

**DOI:** 10.3389/fnmol.2016.00059

**Published:** 2016-07-22

**Authors:** Nickolai V. Vysokov, John-Paul Silva, Vera G. Lelianova, Claudia Ho, Mustafa B. Djamgoz, Alexander G. Tonevitsky, Yuri A. Ushkaryov

**Affiliations:** ^1^School of Pharmacy, University of KentChatham, UK; ^2^Division of Cell and Molecular Biology, Imperial College LondonLondon, UK; ^3^Department of Translational Oncology, P.A. Hertzen Moscow Oncology Research Institute, National Center of Medical Radiological ResearchMoscow, Russia

**Keywords:** Lasso, teneurin-2, cell surface receptor, dimerization, proteolysis, protein processing, shedding, latrophilin-1

## Abstract

Teneurins are large cell-surface receptors involved in axon guidance. Teneurin-2 (also known as latrophilin-1-associated synaptic surface organizer (Lasso)) interacts across the synaptic cleft with presynaptic latrophilin-1, an adhesion G-protein-coupled receptor that participates in regulating neurotransmitter release. Lasso-latrophilin-1 interaction mediates synapse formation and calcium signaling, highlighting the important role of this trans-synaptic receptor pair. However, Lasso is thought to be proteolytically cleaved within its ectodomain and released into the medium, making it unclear whether it acts as a proper cell-surface receptor or a soluble protein. We demonstrate here that during its intracellular processing Lasso is constitutively cleaved at a furin site within its ectodomain. The cleaved fragment, which encompasses almost the entire ectodomain of Lasso, is potentially soluble; however, it remains anchored on the cell surface via its non-covalent interaction with the transmembrane fragment of Lasso. Lasso is also constitutively cleaved within the intracellular domain (ICD). Finally, Lasso can be further proteolytically cleaved within the transmembrane domain. The third cleavage is regulated and releases the entire ectodomain of Lasso into the medium. The released ectodomain of Lasso retains its functional properties and binds latrophilin-1 expressed on other cells; this binding stimulates intracellular Ca^2+^ signaling in the target cells. Thus, Lasso not only serves as a *bona fide* cell-surface receptor, but also as a partially released target-derived signaling factor.

## Introduction

Teneurins are large cell-surface receptors implicated in neuronal migration and development, and axonal guidance (Otaki and Firestein, 1999; Drabikowski et al., 2005; Leamey et al., 2007; Young et al., 2013). There are four homologous teneurin genes in mammals (*TENM1–4*), two in *Drosophila* (*ten-a* and *ten-m*) and one in *Caenorhabditis elegans* (*ten-1*, Tucker and Chiquet-Ehrismann, 2006; Young and Leamey, 2009). Teneurins were independently discovered several times on the genetic level and given different names. Initially, *ten-a* was identified in *Drosophila* in a low-stringency cDNA screening for homology to the extracellular matrix protein tenascin-C (Baumgartner and Chiquet-Ehrismann, 1993); however, the low sequence similarity (35%) was only confined to the conserved epidermic growth factor (EGF) repeats. The *Drosophila* *ten-m* gene was separately identified in two laboratories as a gene regulating body segmentation (pair-rule gene) and termed *ten-m* (Baumgartner et al., 1994) or “odd Oz” (Levine et al., 1994), although it was later proven to be unconnected to embryonic segmentation defects (Zheng et al., 2011). Rat *Tenm2* gene was found in a search for homology to olfactory receptor F5 (Otaki and Firestein, 1999), but turned out to be unrelated to olfactory receptors and was termed neurestin. *Teneurin-4* gene was identified as one of mouse genes upregulated in response to endoplasmic reticulum stress and called DOC4, for “downstream of CHOP, 4” (Wang et al., 1998).

Finally, while studying the functions of a presynaptic Adhesion G-protein-coupled receptor, latrophilin-1 (Lelianova et al., 1997), systematic name ADGRL1 (Hamann et al., 2015), we predicted the existence of a postsynaptic receptor that would provide postsynaptic binding sites for latrophilin-1 and thus organize its distribution in the presynaptic membrane (Volynski et al., 2004). This protein, provisionally termed latrophilin-1-associated synaptic surface organizer (Lasso; Silva et al., 2009a,b), was later isolated from rat brain on a latrophilin-1 column and identified as a splice variant of teneurin-2 (Silva et al., 2011). Thus, Lasso became the only teneurin to be identified at the protein level, in a direct search for its predicted function. Given this protein’s lack of a meaningful similarity to tenascin-C, its high-affinity for latrophilin-1 (Silva et al., 2011; Boucard et al., 2014), and the emerging role of teneurins in synaptic organization (Mosca, 2015), we will refer to this protein here as Lasso.

The functions of teneurins are only beginning to be understood. Lasso/teneurin-2 is highly abundant in the brain, especially in the hippocampus, but is largely absent from non-neuronal tissues (Otaki and Firestein, 1999; Tucker et al., 2001; Kenzelmann et al., 2008). By separating pre- and post-synaptic membranes, Lasso was shown to localize mostly in the postsynaptic membrane in rat brain (Silva et al., 2011). *Tenm2* knockout in mice leads to defects in axon guidance from retinal ganglion cells to the thalamus, resulting in behavioral abnormalities (Young et al., 2013). Other teneurins have also been implicated in axon guidance. For example, teneurin-3 plays an instructive role in the functional wiring of the vertebrate visual system (Leamey et al., 2007; Antinucci et al., 2013). Knockdown of *ten-1* in *C. elegans* results in high embryonic lethality, while surviving embryos exhibit developmental perturbations in motor axon guidance (Drabikowski et al., 2005).

As a cell adhesion molecule (Mosca, 2015), Lasso/teneurin-2 could mediate interactions between neuronal processes, providing substrate for attachment and/or intracellular signals for neurite extension (Rubin et al., 1999; Drabikowski et al., 2005; Al Chawaf et al., 2007; Beckmann et al., 2013). Obviously, to function in cell adhesion, teneurin-2 must have a binding partner. One possibility is a homophilic interaction between teneurins (Oohashi et al., 1999; Bagutti et al., 2003; Boucard et al., 2014), which was proposed to enable cell-cell adhesion (Rubin et al., 2002), although this was contested later (Boucard et al., 2014). Alternatively, teneurins could engage in asymmetric interactions. Indeed, presynaptic ten-a binds postsynaptic ten-m in *Drosophila* neuromuscular junctions (Mosca et al., 2012). Furthermore, Lasso forms a high-affinity trans-synaptic complex with latrophilin-1 (Silva et al., 2011; Boucard et al., 2014). The Lasso–latrophilin-1 pair is not only able to mediate cell-cell adhesion (Silva et al., 2011; Boucard et al., 2014), but can also facilitate the formation of artificial synapses, with Lasso localizing on the topologically postsynaptic membrane (Silva et al., 2011). This asymmetric distribution of the two proteins suggests that Lasso may provide postsynaptic attachments sites and instructive signals for growth cones, which are known to express latrophilin-1 (Silva et al., 2011).

**Figure 1:**
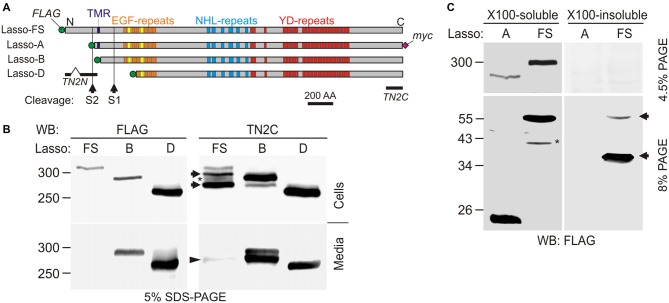
**Latrophilin-1-associated synaptic surface organizer (Lasso) is cleaved inside the cell and partially released into the medium. (A)** A scheme of Lasso constructs used, showing the transmembrane region (TMR), repeated structures in the ectodomain, two proposed cleavage sites and antibody epitopes. AA, amino acids; epitopes recognized by the anti-FLAG, teneurin-2 N-terminal antibody (TN2N), TN2C, and anti-myc antibodies are indicated. **(B)** The processing of Lasso-FS, Lasso-B and Lasso-D in transiently transfected HEK293 cells. Cell lysates and conditioned media were analyzed by sodium dodecyl sulfate polyacrylamide gel electrophoresis (SDS-PAGE) and western blotting (WB) using indicated antibodies. The arrows show two proteolytic fragments of Lasso-FS recognized by the C-terminal antibody only (F290 and F270). Arrowhead indicates fragment F270 released into the medium. Note that the soluble Lasso-B is cleaved within the cells before both of its fragments are secreted. **(C)** Detection of N-terminal fragments produced by furin cleavage of Lasso-FS and Lasso-A expressed in HEK293 cells. Both Triton X-100-soluble and -insoluble fractions were analyzed by SDS-PAGE and WB (the arrows indicate fragments F35 and F56). The data in **(B,C)** are representative of 4–6 independent experiments, which gave similar results. Asterisks in **(B,C)** indicate two complementary fragments (F280 and F40) resulting from proteolysis at a third site.

The structure of Lasso (and all other teneurins) is fully consistent with its role as cell-surface receptor: it is type 2 membrane protein consisting of a cytosolic N-terminus, a single transmembrane region (TMR), and an extracellular C-terminal domain (Figure 1). The large ectodomain contains eight EGF repeats, of which two harbor five (rather than six) cysteines. These unpaired cysteines mediate the covalent homo-dimerization of teneurins (Oohashi et al., 1999; Feng et al., 2002).

However, teneurin-2 appears to be proteolytically cleaved at several positions: (1) within the N-terminal cytosolic domain (Bagutti et al., 2003); (2) near the very C-terminus, producing a so-called teneurin C-terminal-associated peptide (TCAP; Qian et al., 2004); and (3) most unexpectedly, within the C-terminal ectodomain (Rubin et al., 1999; Tucker et al., 2001). Proteolysis at the latter site is thought to be triggered by a homophilic interaction of teneurins between contacting cells, which causes the ectodomain to be released into the medium (Kenzelmann et al., 2007). These findings have led to a considerable uncertainty regarding Lasso/teneurin-2 functions on the cell surface: if Lasso is cleaved upon interacting with its ligands or released by constitutive proteolysis, it simply cannot participate in the maintenance of cell-cell contacts. On the other hand, if Lasso functions in cell adhesion, its ectodomain should not be released into the medium in any significant amounts.

In this study, we investigated the mechanisms of Lasso cleavage, cell-surface anchoring and release. Our findings indicate that ectodomain of Lasso is constitutively cleaved inside the cell but is then non-covalently attached to the cell surface via its transmembrane domain. The ectodomain is partially released in a regulated manner both *in vitro* and *in vivo* due to an additional cleavage within the transmembrane domain. The released ectodomain binds to cells expressing latrophilin-1 and triggers intracellular Ca^2+^ signaling. Thus, the Lasso-latrophilin-1 pair may not only mediate adhesion and signaling interactions between a growing cone and a target dendrite, but may also act as a target-derived soluble ligand and its receptor.

## Materials and Methods

### Materials

All chemicals and reagents were purchased from Sigma-Aldrich, unless otherwise stated. Cell culture reagents were from PAA Laboratories or Life Technologies Ltd. The following primary antibodies were used: rabbit anti-V5 (Sigma-Aldrich, Cat# V8137, RRID: AB_261889); mouse anti-myc (clone 9E10; Sigma-Aldrich, Cat# M4439, RRID: AB_439694); mouse anti-FLAG M2 (Sigma-Aldrich, Cat# P2983, RRID: AB_439685); sheep anti-rat teneurin-2 N-terminal M^1^-K^253^ fragment teneurin-2 N-terminal antibody (TN2N; R&D Systems, Cat# AF4578, RRID: AB_10719438); rabbit anti-human teneurin-2 G^290^-L^358^ fragment teneurin-2 middle fragment antibody (TN2M; Sigma-Aldrich, Cat# HPA038420, RRID: AB_10674682). Mouse polyclonal antibody against the C-terminus of Lasso (dmAb, for consistency termed here TN2C) was made in-house (Silva et al., 2011). Rabbit polyclonal antibody against the III-IV loop of all voltage-gated sodium channels (VGSC) was a gift from M. B. Djamgoz.

### Expression Plasmids

The sequences of human Lasso (Ten-2) mutants used in this study are available at GenBank: Lasso-FS (JF784340), Lasso-A (JF784341), Lasso-B (JF784342), Lasso-D (JF784344). N-terminally tagged rat latrophilin-1 (termed LPH-76) was similar to V5-LPH-A described previously (Volynski et al., 2004), but lacked the C-terminal myc-epitope. All cDNAs were subcloned into the pcDNA3.1 vector (Life Technologies Ltd.).

### Cell Transfection

Cell lines (HEK293A and NB2a) were cultured using standard techniques in DMEM (glutamine, high glucose), supplemented with 10% heat-inactivated FBS (PAA Technologies). Stable cell lines were generated using the Escort III transfection reagent (Sigma-Aldrich) and Geneticin selection (Life Technologies Ltd.). The positive cells were further enriched by fluorescence-assisted cell sorting (BD FACScalibur, BD Biosciences).

### Cells Treatment with DTT or Trypsin

In some experiments, the cells were harvested by gentle trituration in phosphate-buffered saline (PBS) containing 2 mM EDTA and protease inhibitors, and centrifuged. The pellet was resuspended at 1 × 10^6^ cells/ml of PBS or PBS containing 100 mM dithiothreitol (DTT; or 1–100 μg/ml trypsin) and incubated at 37°C for 30 min (15 min with trypsin), with occasional mixing, after which the cells were centrifuged again and the cell pellet was lyzed with Triton X-100 (containing protease inhibitors and EDTA) and centrifuged for 30 min at 10,000× *g* to remove nuclei. The supernatants were analyzed by western blotting (WB) as described below.

### Cells Treatment with EDTA

In some experiments, the cells were cultured to 50–60% confluence, briefly washed with PBS and incubated for 30 min in PBS supplemented with 10 mM EDTA or 5 mM CaCl_2_ and protease inhibitors, with gentle agitation. The supernatants were collected and centrifuged to remove any cell debris, while the cells were lyzed with Triton X-100, as above, and the samples were analyzed by WB.

### Extraction of Cells with Triton X-100

Cultured cells were washed with ice-cold PBS, dislodged using a thin rubber spatula and centrifuged for 10 min at 20,000× *g*, at 4°C. The pellet was freeze-thawed, resuspended in 1% Triton X-100 in PBS, homogenized by passing 20 times through a Hamilton syringe, incubated on ice for 30 min and centrifuged as above. The Triton X-100 soluble supernatant was collected and prepared for WB (see below). The Triton X-100 insoluble pellet was washed with ice-cold PBS by centrifugation, then resuspended in Sample Buffer (see WB), passed 20 times through a Hamilton syringe and sonicated to reduce viscosity. The samples were analyzed by WB.

### Membrane-Bound and Soluble Proteins from Brain

This study was carried out in accordance with the Code of Ethical Practice for Research, University of Kent, and approved by the Research Ethics Advisory Group of the School of Pharmacy. According to standard protocols for brain fractionation (Gordon-Weeks, 1987; Berninghausen et al., 2007), an adult rat brain was homogenized using a Potter-Elvenhejm homogenizer (Sartorius) in 320 mM sucrose, 10 mM HEPES, pH 7.2 and protease inhibitors, and centrifuged for 5 min at 2200× *g* to yield pellet (P1) and supernatant (Sup1). Sup1 was further centrifuged for 20 min at 12,000× *g* to pellet down synaptosomes (P2), while Sup2 was subjected to ultra-high speed centrifugation for 20 min at 100,000× *g*, leaving only soluble proteins in Sup3. The samples were then analyzed by WB, with or without prior treatment with 100 mM DTT.

### Affinity Purification of Proteins

NB2a cells stably transfected with a plasmid encoding LPH-51 or Lasso-D (Silva et al., 2011) were cultured in a serum-free medium for 48 h. Then 25 mL of each medium were centrifuged at 20,000× *g* for 30 min, passed through a 0.2 μm filter and mixed, respectively, with 250 μL of an anti-V5-antibody agarose (Sigma-Aldrich, Cat# A7345, RRID: AB_10062721) or an anti-myc-antibody (AbD Serotec; Cat# MCA2200PE, RRID: AB_2148450) attached to CNBr-activated Sepharose 4B (GE Healthcare). After incubation for 16 h at 4°C, the suspensions were passed through plastic funnels equipped with polypropylene filters (35 μm pore size) and the settled gels were washed with 10 volumes of ice-cold buffer containing 150 mM NaCl, 50 mM Tris-HCl, pH 8.0. The retained proteins were fractionally eluted with 5 × 250 μL of 50 mM triethylamine in 150 mM NaCl (pH ~12), and neutralized with 150 mM NaCl, 1 M HEPES, pH 7.2; the fractions were combined as required, dialyzed overnight against PBS and analyzed by WB. The affinity columns were recovered by extensive washing with PBS and used repeatedly to isolate respective proteins.

Purification of Lasso/teneurin-2 from rat brain was carried out as described previously (Silva et al., 2011). Briefly, rat brains (~5 g) were homogenized in 20 mL of 15 mM HEPES, pH 7.4, 147 mM NaCl, 5.6 mM glucose, 5.6 mM KCl, 1 mM CaCl_2_, 1 mM MgCl_2_ and 1% Triton X-100, using a Potter-Elvenhjem homogenizer. The lysate was incubated on ice for 1 h, centrifuged at 50,000× *g*, and incubated overnight with 250 μL of an anti-V5 antibody column containing 100 μg of pre-adsorbed LPH-51 (purified as described above). The column was washed with 10 volumes of buffer containing 0.3% Triton X-100, then with five volumes of buffer supplemented with 1 M NaCl. Bound proteins were eluted with 5 volumes of 50 mM triethylamine in 150 mM NaCl, 0.3% Triton (pH ~12), and neutralized with 150 mM NaCl, 1 M HEPES, pH 7.2.

### Western Blotting

All samples were dissolved in Sample Buffer, containing 60 mM Tris-HCl, pH 6.8, 6% glycerol, 100 mM DTT (unless specified otherwise) and 2% SDS and treated for 30 min at 50°C. The samples, loaded proportionately to their volume, were separated in 4% to 16% polyacrylamide, Tris-glycine (National Diagnostics, Atlanta, GA, USA) SDS-containing gels. The separated proteins were transferred onto polyvinylidene fluoride membranes (Millipore) in Tris-glycine buffer containing 20% methanol, at 100 V for 90 min (120 min for high MW proteins). The membranes were blocked in 5% non-fat dry milk and stained using respective primary and horseradish peroxidase-conjugated secondary antibodies, followed by chemiluminescent detection using SuperSignal^TM^ West Femto Maximum Sensitivity Substrate (Thermo Scientific) on LAS3000 (FUJIFILM) gel documentation system, with several exposures to determine the linear signal range for each protein band. Where appropriate, the protein bands were quantified using the ImageJ software (NIH, Bethesda, MD, USA; RRID: SCR_003070).

### Mass Spectrometry

Samples for mass spectrometry (MS) were prepared by affinity chromatography, as described above. Lasso-14 from culture medium or Lasso/teneurin-2 from rat brain detergent extract was further separated in a preparative 4% SDS-polyacrylamide gel, which was stained using MS-compatible silver stain (Invitrogen). Protein bands excised from the gel were lyophilized, digested with trypsin (Promega) and analyzed by MALDI-TOF/TOF-MS as previously described (Silva et al., 2011). The data were compared to the Swiss-Prot database (2015). To predict proteolysis sites, Protease specificity prediction server (PROSPER; Song et al., 2012) and ProP 1.0 Server (Duckert et al., 2004) on-line tools were used.

### Immunocytochemistry

Cells grown on poly-D-lysine-coated coverslips were fixed for 10 min with 4% paraformaldehyde, permeabilized with 0.1% Triton-X100 where noted, washed, then blocked for 1 h with 10% goat serum in PBS and incubated with primary antibodies in blocking solution (dilutions used were: anti-myc mAb, 1:1000; anti-V5-mAb, 1:2000) for 1 h at room temperature. The coverslips were then washed 3 times and incubated for 1 h with Alexa-conjugated secondary antibodies in blocking solution, followed by three washes. Coverslips were mounted using FluorSave mounting medium (Calbiochem) and imaged on Zeiss LSM 510 confocal microscope using a 40× oil immersion objective, the proprietary Zeiss software and the same settings for all coverslips. Unadjusted images were quantified using the ImageJ software.

### Intracellular Ca^2+^ Signaling

NB2a cells stably transfected with latrophilin-1 were starved of serum for 24 h, loaded with Fluo-4AM (Thermo Scientific) using the manufacturer’s protocol and equilibrated in Recording buffer (in mM: 145 NaCl, 5.6 KCl, 5.6 glucose, 1 MgCl_2_, 15 HEPES; 0.5 mg/ml BSA; pH 7.4). Images were acquired every 5 s using a laser-scanning confocal fluorescent microscope (LSM510, Zeiss) equipped with a water-dipping objective (Achroplan, 20×, Zeiss), 488 nm laser excitation and a 505–550 nm emission filter. After a 200 s baseline recording, the following treatments were added to the recording chamber: (1) 10 nM purified Lasso-D expressed by stably transfected NB2a cells in Recording buffer; (2) Recording buffer; or (3) 2 nM LTX^N4C^ in Recording buffer, and the imaging continued. To synchronize Ca^2+^ signaling, 2 mM CaCl_2_ in serum-free medium was added at 1500 s, followed by 1 nM wild-type LTX at 3000 s. Ca^2+^ fluorescence in individual cells within each field of view was quantified using the LSM510 software and normalized to the baseline.

### Statistical Analysis

Normally distributed data (as tested using Lillefors test, MATLAB, RRID: SCR_001622) were compared using the unpaired two-tailed *t*-test or one-way ANOVA, with *post hoc* Tukey test and correction for multiple comparisons; *p* < 0.05 was considered to indicate statistically significant difference. Significance levels are denoted in the Figures by asterisks: *p < 0.05; **p < 0.01; ***p < 0.001.

## Results

### Posttranslational Proteolysis of Lasso

Due to its large size and proteolytic cleavage at several positions, it is very difficult to study Lasso cleavage *in vivo*. However, similar to its proteolysis *in vivo* (Tucker et al., 2001), the protein is also cleaved *in vitro* (Rubin et al., 1999). Therefore, we studied the cleavage of recombinant full-size Lasso labeled on both termini with antibody epitopes (Lasso-FS; Figure 1A) in HEK293 cells. We found that this protein was proteolyzed at least at two positions (Figure 1B). In addition to the full-size protein of ~315-kDa, detected by both N- and C-terminal antibodies (Figure 1B), two major C-terminal fragments with apparent molecular masses of 290 and 270 kDa were found in the cells (referred to as fragments F290 and F270; Figure 1B, Cells). Interestingly, a proportion of F270 was released into the medium, while F290 was not (arrowhead in Figure 1B, Media). Together with the fragment sizes, this indicated that one cleavage site (S1) was located in the ectodomain and the second cleavage site (S2) in the cytosolic domain, upstream of the TMR (Figure 1A).

To understand the cleavage at S1 better, F270 was compared to two soluble Lasso constructs, of which Lasso-B was larger than F270, while Lasso-D was smaller (Figures 1A,B). As expected, both Lasso-B and -D were secreted (Figure 1B, Media). In addition, Lasso-B was partially proteolyzed, giving rise to F270 fragment, which indicated that Lasso-B contained the S1 site. From molecular masses of Lasso-FS, -B, -D and F270, cleavage site S1 was localized in the ectodomain, ~16 kDa downstream of the TMR and, therefore, likely corresponded to the furin-cleavage site previously identified in teneurin-2 (RERR; Rubin et al., 1999).

Interestingly, Lasso-B was cleaved at S1. Given that this secreted soluble protein was not anchored on the cell surface, it could only be proteolyzed inside the cell (Figure 1B, Cells). Most of the F270 produced as a result of cleavage was released into the medium, together with a small proportion of the uncleaved Lasso-B (Figure 1B, Media). This observation indicated that cleavage at S1 must occur during intracellular processing of Lasso. This is fully consistent with S1 being targeted by furin, an enzyme principally localized in the trans-Golgi network compartments (Misumi et al., 1991; Zheng et al., 1994; Thomas, 2002).

To verify the positions of S1 and S2 in relation to the TMR, we then compared the processing of Lasso-FS with that of another membrane-bound construct, Lasso-A, which was truncated approximately at S2 and had an uncleaved molecular mass of ~290 kDa (Figure 1C). In this experiment, we specifically looked for N-terminal fragments. WB revealed that, in addition to two C-terminal fragments (F290 and F270, Figure 1B), Lasso-FS cells also contained two N-terminal fragments, F35 and F56 (Figure 1C, arrows). These fragments migrated in the gel slightly slower than their predicted sizes (25 and 46 kDa, respectively). Consistently, cells expressing Lasso-A (lacking the S2 site) contained one C-terminal fragment F270 (Figure 1C) and one N-terminal fragment, F26 (Figure 1C), which also migrated slower than its predicted size of 21 kDa. A similar aberrant electrophoretic migration of the N-terminal fragments was reported previously for teneurin-2 (Bagutti et al., 2003) and teneurin-1 (Kenzelmann et al., 2008). The relationship between the number, sizes and subcellular localizations of the N- and C-terminal fragments in Lasso-FS and -A indicated that cleavage site S1 (which gave rise to F56 in Lasso-FS and F26 in Lasso-A) was located within the extracellular domain at the furin site, and that cleavage site S2 (which gave rise to F35 in Lasso-FS) was located in the cytosolic N-terminal domain, upstream of the TMR, and was absent in Lasso-A.

In addition, two minor fragments with complementary masses were observed in the cells expressing Lasso-FS: N-terminal F40 and C-terminal F280 (Figures 1B,C, asterisks). These fragments indicated that Lasso-FS could also be cleaved at a third site (described below).

For a more precise localization of S1, gel-purified F270 fragment of Lasso-A was analyzed by MS after trypsin digestion. Its MS fingerprint contained peptides downstream of the furin site, including a peptide with a mass of 3469.95 (M + H^+^), which corresponded to the N-terminal peptide of F270 (SIQTLVQNEAVFVQYLDVGLWHLAFYNDGK; calculated molecular mass 3468.90). We did not find in the F270 digest any peptides from the ectodomain upstream of the predicted cleavage site. These results confirmed that F270 was produced by cleavage at the furin site S1 (…RERR∣SIQT…).

Interestingly, the N-terminal fragments produced by cleavages at S1 and S2 had distinct solubilities in Triton X-100. Both TMR-containing fragments produced by S1 cleavage (F56 in Lasso-FS and F26 in Lasso-A) were soluble in Triton. On the contrary, F35 (produced by Lasso-FS cleavage at S2 and containing no TMR) was Triton-insoluble and therefore possibly located in the nucleus, as reported previously for the intracellular fragment of teneurin-2 (Bagutti et al., 2003) and teneurin-1 (Nunes et al., 2005; Kenzelmann et al., 2008).

### Cell-Surface Attachment of Lasso

**Figure 2:**
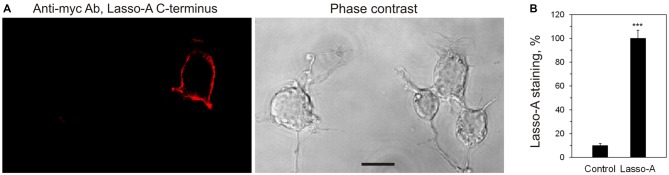
**Lasso is expressed on the cell surface. (A)** Non-permeabilized NB2a cells transiently expressing Lasso-A were fixed, stained for the C-terminal fragment with the primary anti-myc antibody and an Alexa 594-conjugated secondary anti-mouse antibody, and visualized by confocal fluorescent microscopy. Note the surface exposure of Lasso and the lack of staining of non-expressing cells. Scale bar, 10 μm. **(B)** Quantification of Lasso-A staining on the cell surface. Images (as in **A**) were quantified by computer aided densitometry; cells not transfected with Lasso-A were used as control. The data shown are the means ± SD (*n* = 4 independent experiments); the *t*-test compares the staining of Lasso-expressing cells (taken as 100%) to that of untransfected cells; ***p < 0.001.

**Figure 3:**
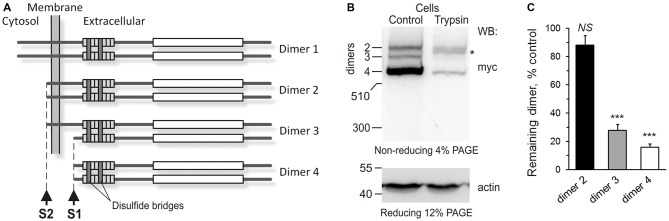
**The anchoring of Lasso ectodomain on the cell surface. (A)** A scheme of Lasso dimers produced by the cleavage of Lasso-FS at sites one cleavage site (S1) and second cleavage site (S2), and the initially proposed mechanism of F270 anchoring on the cell surface. When Lasso-FS (dimer 1) is cleaved at site S2, the resulting dimer 2 is very similar to Lasso-A, and therefore Lasso-A was used in **(B,C)** to study the proposed anchoring of Lasso fragments on the cell surface. **(B)** Cell-surface exposure of Lasso fragments probed by trypsinization. NB2a cells stably expressing Lasso-A were incubated with 1 μg/ml trypsin (Trypsin) or buffer (Control) and analyzed by SDS-PAGE and WB, using anti-myc and anti-actin antibodies. The data are representative of three independent experiments. Asterisk, trypsin-sensitive fragment of the S1-intact Lasso-A (dimer 2). **(C)** Quantification of Lasso-A fragments protection from trypsin. Immunoblots of trypsinized and control cells (as in **B**) were analyzed by quantitative densitometry (after normalization to actin) to determine the amount of each Lasso dimer remaining in the cells. The data shown are the means ± SD (*n* = 3); the *t*-test compares the amount of respective dimer in treated cells to the amount of the same dimer before trypsinization (taken as 100%); ***p < 0.001.

Lasso/Teneurin-2 is a *bona fide* cell-surface receptor (Tucker and Chiquet-Ehrismann, 2006; Silva et al., 2011; Beckmann et al., 2013; Boucard et al., 2014) and can be clearly detected on the cell surface when expressed in HEK293 (not shown) or mouse neuroblastoma NB2a cells (Figure 2). Therefore, the release of its almost entire extracellular domain (F270) into the medium was intriguing. Although the shedding of some cell surface receptors by extracellular matrix metalloproteases is a normal cell surface remodeling process (Nagase et al., 2006; Löffek et al., 2011), Lasso is evidently cleaved inside the cell and thus is presumably destined for secretion rather than turnover. However, a large part of the soluble F270 remained in the cell pellet (Figure 1B, Cells). This could be explained by two possible distribution patterns of Lasso: (1) only the TMR-containing ectodomain (i.e., not cleaved at S1) is exposed on the cell surface, while F270 is either intracellular or secreted; or (2) at least some amount of the cleaved F270 is somehow also anchored on the cell surface: for example, by forming semi-cleaved dimers (Figure 3A, dimer 3, see below).

To determine which Lasso species were exposed on the cell surface, we expressed Lasso-A in NB2a cells, which are similar to neurons that natively express Lasso/Teneurin (Young and Leamey, 2009; Silva et al., 2011). We then treated live cells expressing Lasso-A with trypsin and analyzed the cell pellet by SDS-gel electrophoresis under non-reducing conditions (to prevent the Lasso dimers from breaking up; Figure 3B). We found that the full-size, uncleaved Lasso-A was largely (88 ± 7%, *n* = 3, Figures 3B,C) protected from trypsin and only suffered proteolysis at one specific position that produced a slightly smaller fragment (asterisk in Figure 3B). This indicated that most of the S1-intact Lasso-A was localized inside the cell, in the endoplasmic reticulum and Golgi complex, although some amount of trypsin was able to gain access to it in the trans-Golgi network via the endosomal pathway. In contrast and to our surprise, as much as 84 ± 2% of Lasso fragment F270 was degraded by trypsin (Figures 3B,C). These findings clearly indicated that the vast majority of F270, although being produced inside the cells, was in fact exposed on the cell surface, while the uncleaved protein was only partially exposed or not exposed at all.

How could F270, which had no TMR, be anchored on the cell surface? One mechanism of such anchoring, as mentioned above, could be the dimerization of cleaved Lasso-A fragments with uncleaved, membrane-anchored Lasso-A molecules. Indeed, full-size teneurin-2 is known to form disulfide-linked dimers (Oohashi et al., 1999; Feng et al., 2002), as shown in Figure 3A (dimer 1). Two uncleaved Lasso-A monomers would then make type 2 dimers, with two membrane anchors, while two F270 fragments with no membrane anchors would make a type 4 dimer (Figure 3C). However, if only one monomer in a dimer was cleaved (dimer 3), then F270 would remain on the cell surface, being anchored via the TMR-containing monomer. Indeed, such a semi-anchored dimer was found in the NB2A cells expressing Lasso-A (Figure 3B, dimer 3); this dimer was practically fully accessible to trypsin (~84% lost to trypsin) and, therefore, mostly exposed on the cell surface.

**Figure 4:**
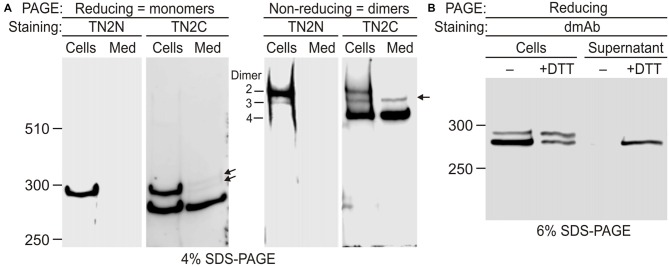
**Disulfide-mediated homodimerization of Lasso. (A)** Lasso-A forms three types of dimers. NB2a cells stably expressing Lasso-A and the conditioned medium were analyzed by SDS-PAGE in reducing and non-reducing conditions and subsequent WB with TN2N (N-terminal) and TN2C (C-terminal) antibodies. Lasso dimers (types 2–4) are indicated on the non-reducing gel. Note that dimers 3 and 4 are partially released into the medium; the released dimers 3 and their monomers (arrows) are not recognized by the N-terminal antibody. **(B)** Lasso fragment F270 is anchored on the cell surface and released by reducing conditions. Live NB2a cells stably expressing Lasso-A were treated with 100 mM dithiothreitol (DTT) and analyzed by SDS-PAGE under reducing conditions, together with the supernatant after DTT treatment. Note that DTT specifically releases a large proportion of the surface-exposed F270. The data in **(A,B)** are representative of three respective independent experiments, which gave similar results.

The mechanisms of Lasso-A secretion and surface anchoring were then studied in detail in NB2a cells, using N- and C-terminal antibodies (Figure 4). In contrast to HEK293 cells, a much larger proportion of Lasso-A (78 ± 4%; *n* = 3) was cleaved at S1 in NB2a cells (Figure 4A, Reducing, Cells), and 55 ± 4% of this F270 was secreted into the medium (Figure 4A, Reducing, Med). To demonstrate the disulfide-mediated Lasso dimerization and the existence of type 3 dimers, the cell extract and conditioned medium were analyzed by SDS-gel electrophoresis in non-reducing conditions. All Lasso-A was found to be in disulfide-linked dimers (Figure 4A, Non-reducing) and all three types of predicted Lasso-A dimers (dimers 2, 3 and 4 in Figure 3C) were identified in the cell pellet (Figure 4A, Non-reducing). About 20% of all Lasso-A in the cell pellet was not cleaved and formed dimer 2 with the molecular mass of ~580 kDa (Figure 4A, Non-reducing, Cells). Type 3 dimers, consisting of one F270 and one full-size monomer (molecular mass of ~560 kDa), were also found. Similar to the results in Figure 3, the semi-cleaved dimers constituted ~10% of cellular Lasso-A. Finally, about 70% of Lasso-A existed in the cells in the form of type 4 dimers (molecular mass 540 kDa), in which both monomers were cleaved at S1 (Figure 4A, Non-reducing, Cells). When released into the medium, F270 also remained in the form of type 4 dimer (Figure 4A, Non-reducing, Med).

To further prove that the semi-cleaved Lasso-A dimer 3 existed on the cell surface, we treated live cells with DTT (Figure 4B). The resulting DTT extract contained F270. This fragment was not released by the cells in the absence of DTT and at least partially came from the reduction of disulfide bridges in semi-anchored dimer 3. The amount of F270 on the cells decreased proportionately (Figure 4B).

These results provided unequivocal evidence for the exposure of F270 on the cell surface and the presence of semi-anchored type 3 dimers, but also highlighted some inconsistency in our original hypothesis that the cell-surface F270 is only anchored via an uncleaved monomer. Indeed, dimer 3 constituted only ~10% of all Lasso-A dimers (Figures 3B, 3B, 4A), while a much larger proportion of the F270 fragment was exposed on the cell surface (84%; Figures 3B,C) and could not, therefore, be anchored via the full-size monomer only. Moreover, most of the surface-exposed F270 was engaged in type 4 dimers (Figures 3B,C), in which none of the monomers had a TMR. This led us to propose that F270 must also be anchored via a different mechanism.

**Figure 5:**
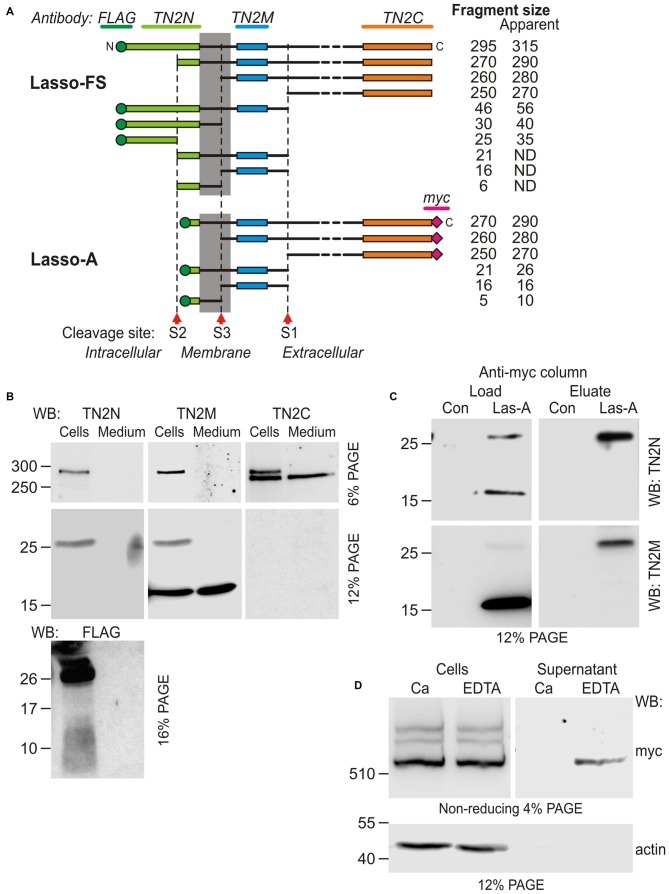
**Additional cleavage and shedding of Lasso ectodomain. (A)** Schematic representations of Lasso-FS and Lasso-A and their fragmentation by proteolysis at sites S1, S2 and S3. Antibody epitopes are shown by different colors; the calculated molecular masses of the resulting fragments are shown on the right; the apparent fragment sizes (including any posttranslational modifications identified by SDS-PAGE) are also indicated (ND, not determined). **(B–D)** Analysis of Lasso-A cleavage by WB. **(B)** Detection of the fragments produced by cleavage at sites S1, S2 and S3. Lasso-A expressing cells and conditioned medium were analyzed by SDS-PAGE and WB using the FLAG, TN2N, teneurin-2 middle fragment antibody (TN2M) and TN2C antibodies. **(C)** The transmembrane fragment F26, but not F16, co-purifies with F270. Extracts from Lasso-A-expressing (or control untransfected) cells were passed through anti-myc-antibody columns and the eluates were analyzed by SDS-PAGE and WB. **(D)** Attachment of Lasso-A ectodomain to the cell surface is sensitive to the removal of divalent cations. Cells expressing Lasso-A were washed and incubated in a Ca^2+^-containing buffer or in 10 mM EDTA, and the high-speed supernatants were analyzed by SDS-PAGE and WB, together with the cell pellets, using anti-myc and anti-actin antibodies. Note that some F270 is lost from the cell surface in the absence of Ca^2+^. The data in **(B–D)** are from one of 2–3 respective independent experiments, which gave similar results.

While analyzing the processing of Lasso, we noticed its multiple similarities with Notch protein and proposed that, similar to Notch, the furin-cleaved F270 fragment of Lasso/teneurin-2 could be anchored on the cell surface via its TMR-containing fragment and partially shed into the medium due to an extra cleavage in or near the TMR of the anchoring fragment. This hypothesis was supported by the finding that a heterodimer similar in size to the membrane-anchored dimer 3 was released into the medium (Figure 4A, Non-reducing, Media). This dimer corresponded to the disulfide-linked complex of F270 with a ~280-kDa fragment. The latter was observed in reducing gels (arrows in Figure 4A, Reducing, Media). Although this fragment had a molecular mass similar to that of membrane-anchored monomer of dimer 3 (~286 kDa), it was not detected by the N-terminal antibody TN2N (Figure 4A, Media, Reducing and Non-reducing) and therefore must have been further cleaved within or outside the TMR, at a third proteolysis site, S3. This was in fact consistent with the observation of additional fragments (N-terminal F40 and C-terminal F280) in our initial experiments with Lasso-FS (asterisks in Figures 1B,C). A map predicting all the fragments that should result from the cleavage of Lasso-FS and -A at the three proteolysis sites is presented in Figure 5A. Most of these fragments were identified in the experiments described above. However, the TMR-containing fragment F26 (Figure 5A; which could provide anchoring for the fully cleaved Lasso-A, dimer 4), and the S3-cleaved fragment F16 were separated from the F270 on an sodium dodecyl sulfate polyacrylamide gel electrophoresis (SDS-PAGE) due to their non-covalent interaction.

To detect the F26 and F16 fragments, we used an antibody (TN2M) against a 68-residue teneurin-2 sequence located between the TMR and the S1 site. This antibody clearly detected in the cell pellet the uncleaved Lasso-A and the F26 fragment (Figure 5B, TN2M), which were also recognized by the N-terminal antibodies (TN2N and FLAG; Figure 5B and also Figure 1C, TN2N, FLAG). In addition, TN2M also stained a fragment of ~16 kDa, which was present both in the cells and in the medium, but was not recognized by the N-terminal antibodies. Reciprocally, the N-terminal FLAG antibody detected a ~10-kDa N-terminal fragment in the cell pellet (Figure 5B, FLAG). Thus, the N-terminal F26 of Lasso-14 was most likely further cleaved at an S3 site, producing the intracellular F10 and the poorly membrane-anchored F16. Given the likely anchoring of F270 on the cell surface via F26, both F26 and F16 would be expected to remain associated with F270 under non-denaturing conditions. To demonstrate this, we performed anti-myc-affinity chromatography of the F270 from solubilized cells and analyzed the eluate by WB. As Figure 5C clearly demonstrates, F26 co-purified with F270, while F16 did not. This confirmed the strong interaction between F26 and F270 and suggested that F16 lost its affinity for F270 (due to a conformational change or a further cleavage).

Potential cleavage site S3 was identified by MS-fingerprint of the gel-purified F16, where a peptide was found with a calculated MW of 2816.44, which corresponded to the tryptic peptide (IAMHLLGLNWQLQPADGHTFNNGIR) between the potential cleavage site and a trypsin-sensitive R residue. A modern tool for prediction of specific protease sites, PROSPER (Song et al., 2012), indicates that this peptide can be generated by a matrix metalloproteinase (MMP).

The non-covalently attached ectodomain of Notch is known to be partially released by chelation of Ca^2+^ (Rand et al., 2000). Likewise, we found that the treatment of NB2a cells expressing Lasso-A with EDTA also facilitated the shedding of some amount of dimerized F270 (Figure 5D). This effect was not due to the activation of an MMP responsible for the cleavage of the anchoring fragment F26, because chelation of Zn^2+^ by EDTA would inhibit MMPs. This further indicated that the interaction between the membrane-anchored Lasso fragment and the furin-cleaved F270 is non-covalent and is partially divalent cation-dependent.

Finally, teneurins have been proposed also to be cleaved at the C-terminus, to produce the biologically active peptide TCAP (Wang et al., 2005; Chand et al., 2013). However, this cleavage was not detected in our experiments with Lasso expression in HEK293 and NB2a cells.

### Regulation of Lasso Cleavage and Release

**Figure 6:**
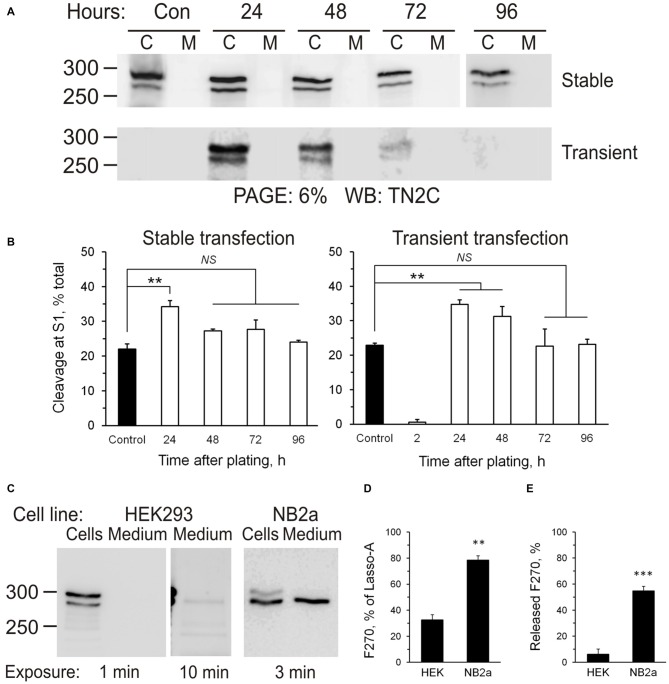
**Regulation of Lasso proteolysis. (A)** Cells maintain the same level of Lasso cleavage at S1. HEK293 cells stably expressing Lasso-A were trypsinized and re-plated or wild-type HEK293 cells were transiently transfected with Lasso-A. The amount of total and cleaved Lasso-A was assessed by SDS-PAGE and WB at different time-points after replating or transfection (C, cells; M, medium; sample loading: 3 × 10^6^ cells/well and equivalent amount of medium/well; Control, a 7-day stable culture of Lasso-A HEK293 cells). The data are representative of three independent experiments. **(B)** Quantification of the data in **(A)**. The data are the means ± SD (*n* = 3); 1-way ANOVA with *post hoc* Tukey test compares the amount of F270 in control to that at different expression stages; **p < 0.01; *NS*, non-significant. **(C)** Differential processing of Lasso at S1 and S3 sites in different cell lines. HEK293 and NB2a cells stably transfected with Lasso-A, and the conditioned media, were analyzed by SDS-PAGE and WB, with the exposure times indicated. The blots are representative of five independent experiments. **(D,E)** Quantification of S1 cleavage and F270 release in different cell lines. Blots (as in **C**) were quantified and the amounts of F270 produced and released were expressed as percentages of all Lasso-A or of all F270, respectively. The data are the means ± SEM (*n* = 5); the *t*-test compares respective values in HEK293 and NB2a cells; **p < 0.01; ***p < 0.001.

Intracellular processing of the ectodomain of Lasso suggested that its cleavage at S1 was constitutive, i.e., occurred continuously during protein processing rather than in response to extracellular signals, for example, Lasso ligands. This idea was born out by the observation that cells produced fairly constant amounts of S1-cleaved Lasso-A (fragment F270), which did not depend on cell density or the amount of expressed Lasso (Figure 6A). S1 cleavage only increased transiently when the cells endured stress (transfection or re-plating during subculture), but then it always returned to the pre-stress value (Figure 6B). Interestingly, different cell lines displayed different S1 activities: thus, 24 h after transfection, HEK293 cells cleaved 36 ± 5% of all expressed Lasso-A, while NB2a cells proteolyzed as much as 78 ± 4% (Figures 6C,D). Similar to HEK293 cells, NB2a cells maintained its characteristic proportion of S1 cleavage, independently of cell density.

Proteolysis at S3 led to the release of Lasso-A ectodomain into the medium. It also appeared to be independent of cell density. Similar to S1, the activity of S3 protease was different in different cell lines. For example, the neuron-like NB2A cells released a disproportionately larger amount of its ectodomain: NB2a shed 55 ± 4%, while HEK293 released only 6 ± 3% of the F270 fragment produced (Figure 6E).

### Lasso Processing in the Brain

**Figure 7:**
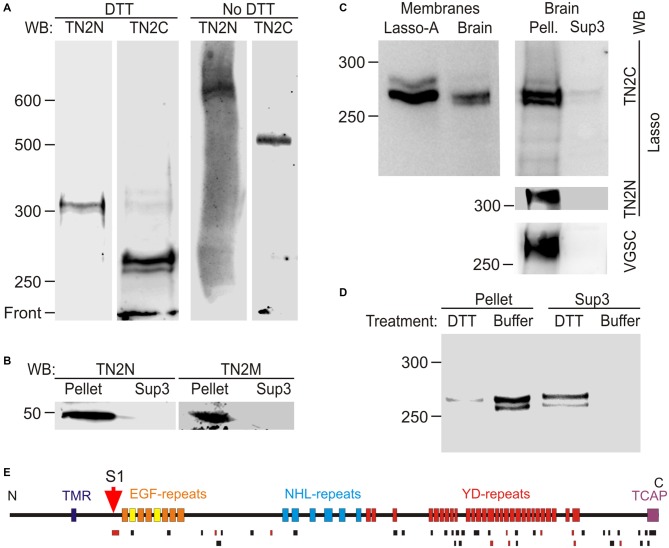
**Proteolytic cleavage and shedding of Lasso *in vivo*. (A)** Lasso in adult rat brain. N- and C-terminal staining of Lasso in rat brain synaptosomes, after SDS-PAGE in reducing and non-reducing conditions. **(B)** Identification of the N-terminal fragment produced by S1 cleavage. The P2 pellet (Pellet) and high-speed supernatant Sup3 fractions from rat brain homogenate were analyzed by SDS-PAGE and immunoblotting. **(C)** Similar processing and behavior of Lasso in brain and cell lines. Left, comparison of cell-attached Lasso in rat synaptosomes and in Lasso-A-expressing NB2a cells. Right, the high-speed supernatant Sup3 from rat brain homogenate contains released S1-cleaved Lasso; staining for Lasso-FS and voltage-gated sodium channels (VGSC) was used to prove the removal of membranes from Sup3. **(D)** Non-covalent attachment of S1-cleaved Lasso to neuronal membranes. Treatment of synaptosomes with DTT (but not with buffer) released almost all cleaved Lasso. The data in **(A–D)** are representative of 2–4 independent experiments, which gave very similar results.** (E)** Mass spectrometry (MS) analysis of Lasso purified from rat brain by latrophilin-affinity chromatography. Top, a schematic map of Lasso, showing cleavage site S1; bottom, peptides identified by MS (black bars) or sequenced by tandem MS-MS (red bars). Note the absence of peptides from Lasso fragments upstream of the S1 site.

It could be argued that the cleavage of Lasso in overexpressing cultured cells might be different from its physiological processing in native tissues. Therefore, we investigated Lasso/teneurin-2 processing in rat brain (Figure 7). Western blot analysis of isolated rat brain synaptic terminals (synaptosomes) demonstrated the presence of two C-terminally stained fragments (268 and 262 kDa; Figure 7A, DTT, TN2C), which were not recognized by TN2N antibody and thus represented native fragments (NF) of Lasso, most likely cleaved at S1. The largest of these fragments, NF268, was slightly smaller than the F270 produced by the cleavage of Lasso-A, because it lacked the two myc epitopes present in Lasso-A (Figure 7A, DTT and 7C, Membranes). Consistent with Lasso/teneurin-2 cleavage at S1, both the N-terminal antibody (TN2N) and the anchoring-fragment antibody (TN2M) recognized a fragment of about 50 kDa (Figure 7B), which represented the whole N-terminal (TMR-containing) fragment upstream of the S1 site (equivalent to F56 in Lasso-FS). Full-size Lasso with a molecular mass of ~310 kDa was also detected in the synaptosomes using the N-terminal antibody (Figure 7A, DTT, TN2N). However, TN2C antibody poorly recognized the full-size Lasso, suggesting that it was present in very small amounts in synaptic membranes compared to transfected HEK cells (Figures 1B,C).

Electrophoresis in non-reducing conditions showed that the native C-terminal fragments (NF268 and NF262) formed dimers, similar to those in cell cultures, that migrated as 535 and 525 kDa bands, respectively (Figure 7A, No DTT). The full-size Lasso (stained with TN2N) formed homodimers migrating at ~620–630 kDa. Interestingly, both the fragments and the full-size Lasso migrated as doublets, in which the two protein bands consistently differed by ~5 kDa under reducing conditions and by ~10 kDa under non-reducing conditions. This similarity suggested that the two bands were likely produced by differential splicing within the ectodomain and that each splice isoform formed homodimers only.

To further characterize Lasso proteolysis in the brain, homogenized rat brain was separated into membrane-bound and soluble proteins by high-speed centrifugation. To control for the complete removal of membrane proteins from the soluble fraction (Sup3), the latter was stained for VGSC and the full-size Lasso. The native Lasso fragments NF268 and NF262 were largely found in the cellular pellet (Figure 7C, Brain, Pell.), but were also present in the high speed supernatant (Figure 7C, Brain, Sup3). These results suggested that, similar to NB2a cells, neurons partially released S1-cleaved Lasso into the extracellular space.

If NF268 and NF262 were attached to neuronal cell surface similar to F270 anchoring in NB2a cells, then they would be expected to be released by disulfide bonds reduction which disrupts the protein folding and dimerization (see Figure 5). Indeed, when the high-speed brain pellet was treated with DTT and centrifuged again, almost all NF268 and NF262 were released into solution (Figure 7D, Sup3, DTT and Pellet, DTT), confirming that these fragments lacked the TMR and were held on the cell surface by non-covalent interactions with the TMR containing fragment (NF50).

To ascertain that NF268 and NF262 were cleaved at the furin site, we purified these fragments using a latrophilin-1 column and SDS-gel electrophoresis. MS analysis of the native Lasso fragments (Figure 7E) revealed a large number of peptides from the ectodomain, including the peptide (MW 3469.95, see above) localized immediately downstream of the S1 (furin) site, but no peptides from the other Lasso domains. Two tryptic peptides were found within the sequence corresponding to TCAP, which indicated that at least the larger of the two NF (NF268) was not cleaved at the C-terminus, contrary to what was proposed previously for teneurin-2 (Chen et al., 2013).

Thus, Lasso is processed by proteolysis and is partially released not only in cultured cells, but also in the brain.

### Released Lasso Binds to Cell-Surface Latrophilin-1

What can be the biological consequence of the shedding of Lasso ectodomain? This protein could diffuse away from the site of release and interact with some cell-surface receptors of other cells, potentially inducing signaling events. Although the released soluble Lasso could engage in homophilic interactions with cell-surface Lasso, its binding to presynaptic latrophilin-1 appears to be stronger (Silva et al., 2011; Boucard et al., 2014).

**Figure 8:**
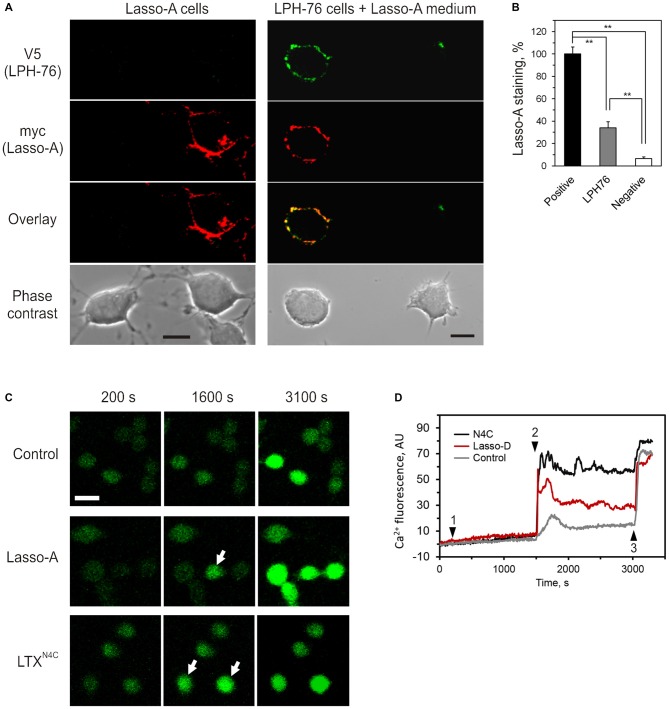
**Released ectodomain of Lasso interacts with latrophilin-1 expressed on other cells**. **(A)** NB2a cells stably expressing Lasso-A were grown for 24 h, the medium harvested by high-speed centrifugation and incubated for 30 min with NB2a cells stably expressing FS latrophilin-1 (construct LPH-76 with an N-terminal V5 tag; see under “Materials and Methods” Section). Both cell cultures were simultaneously fixed without permeabilization and immunostained with rabbit anti-V5 and mouse anti-myc primary antibodies and respective Alexa-conjugated secondary antibodies. The cells were imaged by laser-scanning confocal microscopy, using the same settings. Note the specific binding of soluble released Lasso-A to latrophilin-expressing cells only. Scale bars, 10 μm. **(B)** Quantification of data in **(A)**. The fluorescent signal from positive (Lasso-A expressing), LPH-76 expressing and negative (untransfected) cells was normalized between that of the positive cells and the background without cells in the images. The data (the means ± SEM; *n* = 4) are compared using 1-way ANOVA with *post hoc* Tukey test; **p < 0.01. **(C)** Soluble Lasso ectodomain induces Ca^2+^ signaling in latrophilin-expressing cells. NB2a cells expressing FS latrophilin-1 and loaded with Fluo-4 were imaged in the absence of extracellular Ca^2+^. Buffer (negative control), 10 nM Lasso-D, or 2 nM LTX^N4C^ (positive control) were added to the imaging chamber at time point 1, followed by 2 mM Ca^2+^ at time point 2 and wild-type LTX (1 nM) at time point 3. Arrows indicate cells showing intracellular Ca^2+^ changes after time point 2. Scale bar, 20 μM. **(D)** Cytosolic Ca^2+^ changes in individual cells treated with buffer, Lasso-D or LTX^N4C^. In all experiments (*n* = 3), upon the addition of extracellular Ca^2+^, Lasso-D triggered a strong rise in cytosolic Ca^2+^ in many latrophilin-expressing cells, while the buffer did not.

We, therefore, investigated whether the ectodomain of Lasso released into the medium could bind latrophilin-1. For this purpose, the medium containing released ectodomain of Lasso-A (Figure 4A) was added to cells expressing latrophilin-1. Latrophilin-1-positive cells were identified by immunostaining of the V5 epitope at the N-terminus of recombinant latrophilin-1 (Silva et al., 2009a). Lasso fragment was only found to bind to cells expressing latrophilin-1 (Figure 8).

Moreover, the released soluble ectodomain of Lasso was able to stimulate intracellular Ca^2+^ (${\text{Ca}}_{\text{i}}^{2+}$) signaling in NB2a cells stably expressing latrophilin-1 (Figure 8C). After adding the Lasso-A-conditioned medium (or LTX^N4C^, used as a positive control), Ca^2+^ signaling was synchronized in all cells by adding extracellular Ca^2+^ (${\text{Ca}}_{\text{e}}^{2+}$). Wild type LTX was added at the end of recording to identify cells capable of latrophilin-mediated signaling. Addition of ${\text{Ca}}_{\text{e}}^{2+}$ led to a sharp increase in ${\text{Ca}}_{\text{i}}^{2+}$ only in latrophilin-expressing cells pre-treated with LTX^N4C^ or soluble Lasso fragment (Figure 8D). This rise in ${\text{Ca}}_{\text{i}}^{2+}$ was most likely due to store-operated Ca^2+^ entry induced by latrophilin-1-mediated signaling and did not occur in untransfected cells within the same cultures or in the latrophilin-expressing cells treated with buffer (control).

## Discussion

**Figure 9:**
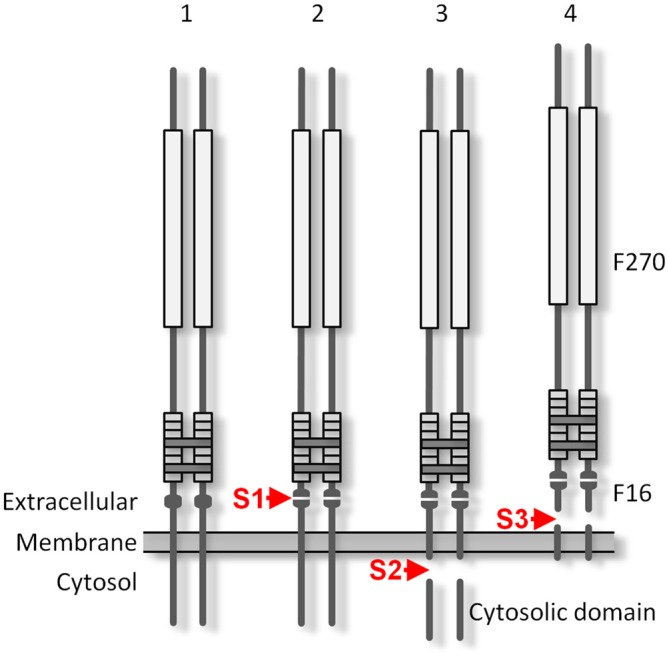
**The proteolytic processing of Lasso.** The protein is produced as a disulfide-linked homodimer (1) and is cleaved at the furin cleavage site S1 during its processing in the Golgi complex (2). The cleaved F270 fragment remains non-covalently attached to the cell surface via the two anchoring domains that contain TMRs. As a result of intracellular proteolysis at site S2 (3), the cytosolic tails of the anchoring domains are released and translocate to the nucleus. Cleavage by an matrix metalloproteinase (MMP) at S3 (4) releases the whole ectodomain of Lasso. Constitutive S1 cleavage is very efficient in the brain and may be enhanced by cell stress; S2 proteolysis may occur in overexpressing cultured cells, but was not observed in rat brain synapses; the extent of S3 cleavage depends on the cell type.

The data presented in this study show a complex molecular architecture and post-translational processing of Lasso/Teneurin-2 (Figures 5, 9). We have confirmed that this protein has an intracellular N-terminus, a single TMR and a large C-terminal ectodomain, and exists as a disulfide-linked homodimer. Lasso can be proteolytically cleaved *in vivo* and *in vitro* at least at three sites: (1) the furin site (S1) within the proximal part of the ectodomain; (2) the cytosolic site S2 within the N-terminal domain, located close to the TMR; and (3) the intramembrane site (S3), which is likely targeted by an MMP. The individual role of each cleavage sites is clearly different.

The furin-mediated cleavage at site S1 occurs during Lasso processing in the Golgi complex. Site S1 is processed very reliably in cell lines, especially in neuron-like neuroblastoma cells, even when the protein is over-expressed. In mature neurons, the cleavage at this site is essentially complete. Proteolysis at S1 produces a large extracellular fragment, F270, which encompasses almost the entire ectodomain, including the EGF repeats that mediate its dimerization. Most Lasso homodimers contain two cleaved monomers, although in about 10% of molecules, only one monomer is proteolyzed, while the second monomer remains intact and provides a membrane anchor. However, even when both monomers undergo S1 proteolysis, the dimeric ectodomain fragments are not directly released into the medium: they remain non-covalently associated with the N-terminal fragments which contain the TMRs. This association is strong and endures most non-denaturing conditions. For example, chelation of divalent cations in the medium only leads to the dissociation of a small proportion of these complexes. In contrast, reduction of disulfide bonds (between the monomers and within the EGF repeats) in the presence of DTT breaks up most non-covalent attachment complexes (Figures 4B, 7D).

The second proteolytic processing (S2) occurs within the cytosolic domain of Lasso when it is over-expressed in cell lines. This cleavage produces cytosolic fragments; however, these fragments are not soluble in non-denaturing detergents, suggesting that they are either aggregated or translocated to the nucleus, as hypothesized previously (Bagutti et al., 2003). The presence of the cytosolic domain in a Lasso construct decreases its expression by about 5-fold (data not shown). It is possible that homophilic interaction of overexpressed Lasso on the cell surface induces the S2 cleavage and sends a nuclear signal inhibiting transcription of genes important for cell survival. In agreement with this, only a very small amount of FS Lasso/teneurin-2 is found in neurons (Figure 7A). In addition, nerve terminals did not contain any fragments that would be consistent with S2 proteolysis (Figure 7). It is possible that homophilic Lasso complexes that may form on the surface of contacting dendrites downregulate Lasso transcription, providing a mechanism that limits dendro-dendritic interactions in the developing brain. The function of proteolytic processing at S2 and the enzyme involved in it will require further investigation.

In addition, cleavage at another site near the C-terminus of teneurin-3 (Qian et al., 2004) and other teneurins (Wang et al., 2005; Young and Leamey, 2009) has been proposed to produce TCAP, a peptide that is believed to act as a neuromodulator (Wang et al., 2005). However, we have not observed this cleavage *in vitro* or *in vivo*.

Given that S1-cleaved Lasso remains constantly on the cell surface in cell lines and neurons (Figures 3B, 7) and participates in cell adhesion (Silva et al., 2011; Boucard et al., 2014), S1 proteolysis cannot be the mechanism by which Lasso/teneurin-2 ectodomain is released into the medium. Instead, cleavage at S3 site within the Lasso TMR, breaks up the membrane-anchored fragment and releases the whole ectodomain (Figure 9). Proteolysis at S3 occurs independently of S1 cleavage, as evidenced by the release of S1-intact Lasso-A (Figure 4A, arrows). The S3 protease is apparently localized on the plasma membrane, because it only affects Lasso-A dimers 3 and 4 which appear on the cell surface (Figure 3B), but not the intracellular dimers 2 (FS homodimers; Figure 4A). Site S3 is most likely cleaved by an MMP. Interestingly, when the anchoring fragment is cleaved at S3, it loses its affinity for the F270 homodimer (Figure 5C). In neurons, S1 proteolysis is very robust, but S3 cleavage is relatively inefficient. Thus, neurons shed 8 ± 4% of total expressed Lasso-A (Figure 7), while neuroblastoma cells release 46 ± 3% (Figure 4). On the other hand, neurons also express large amounts of latrophilin-1, which strongly interacts with the ectodomain of Lasso (Kd = 0.47–1.7 nM; Silva et al., 2011; Boucard et al., 2014), and some amount of the released soluble ectodomain of Lasso may remain bound to latrophilin-1. Further studies will be required to identify the enzyme/s involved in the cleavage at site S3 and its regulation.

The general structure and cleavage of Lasso uncannily resembles those of Notch, a protein that has important cell-fate signaling functions (Tien et al., 2009; Groot and Vooijs, 2012). Although the membrane topography of the two proteins is opposite (the N-terminus of Notch is extracellular, while the N-terminus of Lasso is cytosolic), both proteins contain multiple EGF repeats in their ectodomains and form dimers (Oohashi et al., 1999; Kelly et al., 2010). Both proteins are cleaved at a furin site within the ectodomain, but remain anchored on the cell surface via a non-covalent interaction with the TMR-containing fragment (Sanchez-Irizarry et al., 2004; Gordon et al., 2007; Figures 3B, 5). Both proteins can be cleaved at a cytosolic site. Finally, MMP-mediated cleavage at a third site near the TMR leads to the shedding of the whole ectodomain in both proteins (Tien et al., 2009; Groot and Vooijs, 2012).

However, Lasso differs from Notch in many respects. Thus, the interaction between the ectodomain and the TMR-containing fragment in Notch is mediated by the LNR repeats, which contain multiple disulfide bonds and bind Ca^2+^ and Zn^2+^ (Gordon et al., 2007). Lasso lacks the LNR repeats, but is sensitive to DTT and EDTA, suggesting that the EGF repeats might play a role in the divalent cation-dependent anchoring of the S1-cleaved ectodomain in Lasso. Also, the shedding of the ectodomain in Notch is induced by proteolysis in response to the binding of its ligands: Delta, Serrate, or Lag2 (Tien et al., 2009; Groot and Vooijs, 2012). In contrast, ectodomain shedding in Lasso, is unlikely to be ligand-induced for several reasons. First, Lasso ectodomain shedding stably occurs in cell cultures of different density (where Lasso-Lasso interactions would be highly variable) and does not require interaction with latrophilin-1. Second, in the brain Lasso is engaged in strong complexes with latrophilin-1 and co-purifies with it (Silva et al., 2011). If such interactions caused Lasso ectodomain shedding, Lasso would be unable to mediate cell adhesion, observed in several publications (Rubin et al., 2002; Beckmann et al., 2013; Boucard et al., 2014). Third, any shed Lasso would remain bound to its ligands (latrophilin-1 and Lasso) and would not be released into the medium (likely followed by endocytosis and degradation). Therefore, Lasso release into the medium is most likely regulated in a different manner and has a different function from that in Notch.

The fact that Lasso/teneurin-2 can be released into the medium by regulated proteolysis provides an intriguing possibility that Lasso might act not only as a cell-surface receptor, but also as a soluble, target-derived factor. Such a function is not unprecedented; for example, amyloid precursor protein (APP) can be cleaved, and the released sAPPα can act as a soluble cue regulating proliferation of neural progenitor cells (Caillé et al., 2004; Lazarov and Demars, 2012). Neural cell adhesion molecule (NCAM) is another classic example of a single-pass transmembrane molecule that normally mediates axonal adhesion, but can undergo cleavage and release, enabling it to act as an axonal attractant (Kalus et al., 2006). Among other cell-surface molecules with established roles in axon guidance, Ephrins can also be cleaved and released from the membrane, acting as target-derived axonal repellants (Himanen et al., 2007).

What could the role of soluble Lasso ectodomain in the brain? Usually, fragments of transmembrane proteins released into the medium perform functions different from those of the parental protein. The interaction between the membrane-bound post-synaptic Lasso and pre-synaptic latrophilin-1 is restricted to the synapse (Silva et al., 2011; Mosca et al., 2012; Boucard et al., 2014), where it can contribute to synapse formation (Silva et al., 2011; Boucard et al., 2014). However, when a soluble ectodomain of Lasso diffuses away and binds latrophilin-1 (Figure 8) expressed on distant axons and growth cones (Silva et al., 2011), it might induce some presynaptic specialization, but will be unable to mediate synapse formation. More likely, released Lasso would probably perform a novel function, likely associated with activation of latrophilin-1, with a potential to regulate a variety of intracellular signaling mechanisms. Indeed, teneurin-2 has been proposed to play a role in axon guidance (Young et al., 2013), by supporting neurite outgrowth (Minet et al., 1999) and enlarging growth cones (Rubin et al., 1999). More specifically, a soluble synthetic peptide corresponding to teneurin TCAP-1 (which is similar across all four teneurin homologs) has been shown to cause neurite fasciculation in cultured hippocampal neurons and increase the length of neurite bundles (Al Chawaf et al., 2007), while a longer fragment of Lasso has been shown to trigger Ca^2+^ transients in synaptic boutons (Silva et al., 2011). The latter mechanism may induce growth cone turning in the direction of Lasso gradient (see Kalil et al., 2011). Indeed, our preliminary data indicate that developing neurons release soluble Lasso, which induces axonal attraction (article in preparation).

Thus, Lasso and latrophilin-1 represent a novel receptor pair that, similar to other synaptic adhesion receptors, mediates both proximal contacts and distant communications between dendritic spines and growth cones. Further studies of this new adhesion receptor pair will bring about new insights into brain development and the early stages of synapse formation.

## Author Contributions

YAU conceived and coordinated the study, analyzed the results and wrote the article. NVV, J-PS, VGL, CH designed, performed and analyzed the experiments. MBD coordinated experiments shown in Figures 3–5. AGT coordinated MS experiments and made important intellectual contributions to the manuscript. All authors were involved in revising the article and approved the final version of the manuscript.

## Funding

This work was supported in part by a Wellcome Trust Grant (WT083199MA) and a research fund from the Medway School of Pharmacy to YAU, and in part by the Russian Scientific Foundation (Grant 14-44-00051 to AGT).

## Conflict of Interest Statement

The authors declare that the research was conducted in the absence of any commercial or financial relationships that could be construed as a potential conflict of interest.

## Abbreviations

AA: amino acids
DTT: dithiothreitol
EGF: epidermal growth factor
F: fragment
FS: full size
ICD: intracellular domain
Lasso: latrophilin-1-associated synaptic surface organizer
LPH: latrophilin-1-derived constructs
MMP: matrix metalloproteinase
NF: native fragment
NCAM: neural cell adhesion molecule
PBS: phosphate buffered saline
SDS-PAGE: sodium dodecyl sulfate polyacrylamide gel electrophoresis
TCAP: teneurin C-terminal-associated peptide
TMR: transmembrane domain
TN2M: teneurin-2 middle fragment antibody
TN2N: teneurin-2 N-terminal antibody
VGSC: voltage gated sodium channel
WB: western blotting.
